# Special Issue: Diagnosis and Management of Addiction and Other Mental Disorders (Dual Disorders)

**DOI:** 10.3390/jcm10061307

**Published:** 2021-03-22

**Authors:** Ana Adan, Marta Torrens

**Affiliations:** 1 Department of Clinical Psychology and Psychobiology, School of Psychology, University of Barcelona, Passeig de la Vall d’Hebrón 171, 08035 Barcelona, Spain; 2 Institute of Neurosciences, University of Barcelona, 08035 Barcelona, Spain; 3 Addiction Research Group (GRAd), Neuroscience Research Program, Hospital del Mar Medical Research Institute (IMIM), 08003 Barcelona, Spain; MTorrens@parcdesalutmar.cat; 4 Institut de Neuropsiquiatria i Addiccions, Hospital del Mar, 08003 Barcelona, Spain; 5 Psychiatry Department, Universitat Autònoma de Barcelona (UAB), Cerdanyola del Vallès, 08093 Barcelona, Spain

The term “dual disorder” (DD) refers to the coexistence or concurrence of at least one substance use disorder (SUD) and another mental disorder in the same person, as the World Health Organization established in its lexicon of alcohol and drug terms [1]. At the beginning of the last decade, the World Psychiatric Association (WPA) created a new section for this topic, deciding to use the term dual disorder/pathology.

DD has been associated with a worse prognosis in affected people. Compared with patients with a single disorder, SUD or another psychiatric disorder, patients with DD show greater psychopathological severity, greater attendance at emergency services, as well as a higher frequency of psychiatric admissions and a higher prevalence of suicide. In addition, DD patients present more risk behaviors, which are linked to infections like HIV and hepatitis B and C, as well as to unemployment, homelessness and illegal behaviors. Thus, taking into account the burden it places on the health and legal systems, psychiatric comorbidity among persons who use drugs carry high costs for society. Studies have shown that comorbid disorders are reciprocally interactive and cyclical, and a poor prognosis can be expected for both SUD and other mental disorders if treatment does not address DD in an integrated way [2,3,4].

DD is common, with different prevalence figures for different combinations of mental disorders and SUDs. The most frequent DDs are depression, anxiety (mainly panic disorder and generalized anxiety disorders), post-traumatic stress disorder, psychotic spectrum disorders, attention deficit disorder with or without hyperactivity, and personality disorders (mainly antisocial and borderline). These are combined with the different SUDs, both for the consumption of legal substances (i.e., tobacco, alcohol) and illegal substances (e.g., heroin, cocaine and methamphetamine).

The etiology and phenotypic expression of DDs adds difficulty to the already complex model of multiple risk and protective factors associated with any of the mental disorders. To date, the correct detection, diagnosis and therapeutic intervention in DD patients is a complicated task and a pending challenge among professionals in the field of mental health and addictions.

Providing effective treatments for DDs is a relevant concern not only because of the clinical and social severity of the patients but also because of the difficulties in accessing and coordinating the services where they receive treatment. Although specialized treatment units are advocated, these are scarce or non-existent, depending on the country. The usual thing is to manage patients either in mental-health care centers or in centers specializing in the treatment of SUDs. In both cases, there is a shortage of DD specialists. The differences in the therapeutic approach are important, but it is not always possible to carry out communication that allows multidisciplinary work between centers.

This special issue aims to contribute to the knowledge of patients with a DD, with new advances that facilitate the development of possible preventive interventions and more individualized therapeutic strategies. For this, we have had the participation of various prestigious DD groups that have contributed to a variety of studies, from neurobiology to psychological and social aspects, which suggests lines for future research.

The most frequent pattern in both SUD and DD patients is polydrug use, which makes it difficult to determine the effect of the different substances and even determine the main substance of dependence in a cross-sectional study. Starting from this reality, prioritizing the influence and differential aspects of comorbid psychiatric disorder, regardless of consumption, seems the most practical approach. Thus, Masiak and colleagues [5] have explored the prevalence of certain polymorphisms in candidate genes associated with dopaminergic receptors and transporters in polydrug users with various comorbid disorders. The most robust result was obtained for DRD4 Ex3 gene polymorphism, with the s/s genotype and the s alleles as more common for comorbid psychotic disorders and generalized anxiety. In addition, the s alleles also appeared more common in comorbid depressive episode and dysthymia. Advances in this line can be expected to crystallize in pharmacogenetic findings for a better approach for patients with DD.

If one substance does deserve special attention for its high worldwide consumption and controversy regarding the benefits and risks of its use, it is cannabis. The Hasin and Walsh review [6] confirms strong evidence for an increased risk of developing a comorbid psychotic disorder and, to a lesser extent, other comorbidities such as mood, anxiety and personality disorders in cannabis users. More research is required in the immediate future on the etiology and course of DD with cannabis use since in therapeutic centers, the prevalence of cases is relevant, and both the approach and the prognosis present differential characteristics.

Comorbidity between SUD and major depression (MD) is the most common DD in the field of substance addiction. Three contributions from this special issue have focused on the study of this comorbidity, which we will refer to as dual depression. The research by Marquez-Arrico et al. [7] found that various dimensions of a health-related quality of life in patients with dual depression are worse than those with only SUD although the presence of depressive symptoms and not DD explains the differences observed in physical functioning and health change. In addition, the quality of life shows the predictive capacity of a relapse at a one-year follow-up differential according to the diagnosis. While physical functioning is sensitive in SUD patients, general health is the indicator in the case of asymptomatic dual depressive patients. The Farre et al. [8] study was aimed at investigating the clinical, biological and genetic source of alcohol-induced depression in respect of depression without an SUD. In patients with alcohol-induced depression, they found differences among groups with a greater family history of alcoholism or other SUD, while patients with only depression showed a greater family history of depression. Also, lifetime stressors like physical abuse, childhood abuse and intimate partner violence, difficulties in concentration and suicidal thoughts were more frequent in patients with alcohol-induced depression than in patients with only depression. However, non-genetic differences were found.

Continuing with the neurobiology of drug-induced depression, the study by Fonseca et al. [9] assessed the tryptophan–serotonin (Trp/5-HT) system by means of the acute tryptophan depletion test (ATD), and the kynurenine pathway in subjects who had cocaine-induced depression, cocaine-primary depression, only depression, or were healthy controls. Interestingly, the results suggested that the neurobiological basis for cocaine-induced depression does not seem to be primarily mediated by 5-HT dysfunction, but is probably more related to other neurotransmitters. Deepening this line of work may mean not only improving the understanding of the neurofunctional aspects of dual depression but also making progress towards a more effective psychopharmacological approach to these patients.

Two other empirical studies explored the existence of indicators of easy clinical evaluation in high prevalence comorbidities such as schizophrenia and post-traumatic stress. Río-Martinez et al. [10] have shown an endophenotype of personality traits characteristic of dual schizophrenia, which is related to more problematic clinical characteristics. The personality traits are modifiable and their consideration may be useful for designing specific intervention strategies. Brunault et al. [11] observed that the existence of childhood trauma is more related to the clinical remission of patients with a diagnosis of alcohol-use disorder and post-traumatic stress disorder than the severity of both diagnoses. Along the same line is the contribution of Blanco et al. [12], which points to childhood maltreatment as a predictor of both developing a DD and a more complex and severe clinical profile. In DD patients, the evaluation of traumatic events in childhood and integrating specific therapy into the treatment is an option for a better prognosis.

The research of Roncero et al. [13] and Luca and Peris [14] provided us with data on the presence of sleep disorders in DD patients, greater than 50%, an area that has very few previous studies. The evidence leaves no doubt about the role of altered sleep patterns (delayed sleep induction, sleep fragmentation, early awakening) in low sleep quality, which is more severe in outpatients and in those with comorbid depression. Incorporation in the diagnostic evaluation of sleep disturbances seems mandatory and both studies show us that it can be performed in a reliable and valid way with standardized self-applied or hetero-applied instruments that do not require a great deal of time or the involvement of specific units for sleep disorders. Detecting the type and magnitude of sleep impairment at the beginning of treatment should lead to the incorporation of chronobiological approach strategies (time habits), which can minimize the need to administer hypnotic or sedative drugs in patients diagnosed with both an SUD and a DD. Successfully addressing sleep disorders improves daytime arousal and mood. This can be positive for alleviating withdrawal symptoms and the symptoms of the comorbid disorder.

Finally, two contributions focus on the current state of the clinical evaluation and management of DD patients. Pacini and colleagues [15] developed a specific hierarchical algorithm to be followed for treating DD in heroin use disorder patients, a complex comorbidity that with different consumption patterns has shown an emerging state in recent years. The review on the quality of the clinical recommendation management guidelines, carried out by Hakobyan et al. [16], suggests the use of the NICE guideline to better meet the standards although it shows a clear need to improve the evidence-based recommendations for integrated treatment in DDs. In this sense, much remains to be done as the United Nations Commission on Narcotic Drugs–World Health Organization (WHO) has recently urged United Nations member states [4].
